# A Phase II Study of Docetaxel in Chemotherapy-Naïve Patients With Recurrent or Metastatic Adult Soft Tissue Sarcoma

**DOI:** 10.1080/13577149878136

**Published:** 1998-03

**Authors:** Vivien Bramwell, Martin Blackstein, Karl Belanger, Shail Verma, Sandra Beare, Elizabeth Eisenhauer

**Affiliations:** 1 London Regional Cancer Centre Ontario Canada; 2 Mt Sinai Hospital Toronto Canada; 3 Hopital Notre-Dame Montreal Canada; 4 Ottawa Regional Cancer Centre Ontario Canada; 5 National Cancer Institute of Canada Clinical Trials Group (NCIC-CTG) Ontario Kingston Canada; 6 Department of Medical Oncology London Regional Cancer Centre 790 Commissioners Road East Ontario London N6A 4L6 Canada

## Abstract

*Purpose:* To determine the efficacy and toxicity of docetaxel as first-line chemotherapy in adult patients with locally advanced and/or metastatic soft tissue sarcoma (STS).

*Patients/methods.* Thirty eligible patients, with histologically proven STS, Eastern Cooperative Oncology Group (ECOG) performance status 0–2 and bidimensionally measurable disease, entered this study. None had received previous chemotherapy. Docetaxel 100 mg m^-2^ was given as a 1-h intravenous infusion every 3 weeks. Patients were evaluable for response, evaluated by WHO criteria, after one cycle of chemotherapy and toxicity was graded by NCIC-CTG common toxicity criteria.

*Results.* One hundred and thirty two cycles were aldministered, with a range per patient of 1–9. The median delivered dose intensity was 32.2 mg m^-2^ weekm^-1^ (planned 33.3 mg m^-2^ weekm^-1^ ) and 67% of patients received ≥90% planned dose intensity. There were three partial responses (10.7%; 95% confidence interval 2.3–28.2) with a median duration of 7 months (range 6.4–8.3 months). Thirty patients were evaluable for non-haematological toxicity and 28 for haematological toxicity (repeat counts were not available in two patients). Haematological toxicity was moderately severe, with 18 (64%) patients experiencing at least one episode of grade 4 neutropenia, and 7 (25%) patients experiencing febrile neutropenia.

*Conclusions.* In this study, activity of docetaxel in adult chemotherapy-naïve patients with advanced STS was modest


*Sarcoma* (1998) **2**, 29–33

ORIGINAL ARTICLE

### A phase II study of docetaxel in chemotherapy-naïve patients with recurrent or metastatic adult soft tissue sarcoma

VIVIEN BRAMWELL,[1] MARTIN BLACKSTEIN,[2] KARL BELANGER,[3] SHAIL VERMA,[4] SANDRA BEARE[5] & ELIZABETH EISENHAUER[5]

[1]*London Regional Cancer Centre, Ontario,* [2]*Mt Sinai Hospital, Toronto,* [3]*Hopital Notre-Dame, Montreal,* [4]*Ottawa Regional Cancer Centre, Ontario &* [5]*National Cancer Institute of Canada Clinical Trials Group (NCIC-CTG), Kingston, Ontario, Canada*

**Abstract**

*Purpose*: To determine the efficacy and toxicity of docetaxel as first-line chemotherapy in adult patients with locally advanced and/or metastatic soft tissue sarcoma (STS).

*Patients/methods.* Thirty eligible patients, with histologically proven STS, Eastern Cooperative Oncology Group (ECOG) performance status 0–2 and bidimensionally measurable disease, entered this study. None had received previous chemotherapy. Docetaxel 100 mg $m^{-2}$ was given as a 1-h intravenous infusion every 3 weeks. Patients were evaluable for response, evaluated by WHO criteria, after one cycle of chemotherapy and toxicity was graded by NCIC-CTG common toxicity criteria.

*Results.* One hundred and thirty two cycles were aldministered, with a range per patient of 1–9. The median delivered dose intensity was 32.2 mg $m^{-2}$ $week^{-1}$ (planned 33.3 mg $m^{-2}$ $week^{-1}$) and 67% of patients received $\geq$90% planned dose intensity. There were three partial responses (10.7%; 95% confidence interval 2.3–28.2) with a median duration of 7 months (range 6.4–8.3 months). Thirty patients were evaluable for non-haematological toxicity and 28 for haematological toxicity (repeat counts were not available in two patients). Haematological toxicity was moderately severe, with 18 (64%) patients experiencing at least one episode of grade 4 neutropenia, and 7 (25%) patients experiencing febrile neutropenia.

*Conclusions.* In this study, activity of docetaxel in adult chemotherapy-naïve patients with advanced STS was modest

**Key words**: *docetaxel, chemotherapy, soft tissue sarcoma*

### Introduction

In the past 10 years, there has been little progress in the management of metastasized soft tissue sarcoma (STS). A minority of patients have long-term benefit from resection of lung metastases.[1] No new active agents have been identified since doxorubicin and dacarbazine in the 1970s, and ifosfamide in the mid-1980s.[2] Despite encouraging data from single-arm studies, randomized trials have failed to demonstrate convincingly the superiority of combination chemotherapy[3] compared with single-agent doxorubicin. Similarly, dose intensification of standard agents or regimens, which have produced promising response rates in pilot studies, have not been proven more active in randomized trials.[4] The need for active new agents is clear.

To optimize the possibility of identifying new active drugs for this disease, the Canadian Sarcoma Group working with the NCIC Clinical Trials Group has pursued for several years, a policy of offering investigational drug treatment to chemotherapy-naïve patients, with the option to proceed to standard drugs if initial treatment fails.

Docetaxel (Taxotere) is a semi-synthetic analogue of paclitaxel, prepared using a precursor extracted from the needles of the European yew, *Taxus baccata*.[5] By enhancing microtubule assembly and inhibiting depolymerization of tubulin, docetaxel causes accumulation of microtubules in the cell and, by blocking cells in the M-phase of the cell cycle, prevents cell division.[6] Based on more potent *in vivo* antitumour activity compared with paclitaxel, in B16 melanoma and a variety of colon carcinomas,[7] as well as a favourable toxicity profile in animals, docetaxel was selected for human studies. Comparing the toxicities observed in the five human phase I trials, the highest maximum tolerated dose (MTD) and dose intensity were achieved with the 1-h 3-weekly infusion schedule.[8] The dose selected for this, and other phase II studies, was 100 mg $m^{-2}$.

Correspondence to: V. Bramwell, Department of Medical Oncology, London Regional Cancer Centre, 790 Commissioners Road East, London, Ontario, Canada N6A 4L6. Tel: +1 519 685 8640; Fax: + 519 685 8624; E-mail: vbramwell@lrcc.on.ca.

1357-714X/98/040029-05 ©1998 Carfax Publishing Ltd

### Patients and Methods

### *Criteria for eligibility*

Patient eligibility criteria included: histologically proven STS; bidimensionally measurable metastatic or locally recurrent disease incurable with standard therapy; ECOG performance status 0, 1 or 2 and life expectancy of 12 weeks or greater; age $\geq 18$ years; no previous chemotherapy; no prior malignancy; granulocytes $>2.0\times10^9$/l, platelets $>100\times10^9$/l, creatinine and bilirubin $\leq 1.5\times$ upper normal limit; signed informed consent. Patients were not permitted to have prior radiation to the sole site of measurable disease.

### *Trial design and therapeutic regimen*

Docetaxel was supplied by Rhône Poulenc-Rorer Ltd (Ville St Laurent, Quebec, Canada). It was given at a starting dose of 100 mg $m^{-2}$, diluted in 250 ml of 0.9% sodium chloride or 5% dextrose solution, infused over 1 h, repeated every 3 weeks. All patients were premedicated for possible hypersensitivity with 20 mg of dexamethasone orally 12 and 6 h prior to docetaxel, and 50 mg of diphenhydramine and 50 mg of ranitidine (or 300 mg of cimetidine) given slowly intravenously prior to docetaxel.

Doses were adjusted for myelosuppression based on nadir blood counts. For an absolute granulocyte count of $<0.5\times10^9$/l for 7 days or a platelet count $<25\times10^9$/l, febrile neutropenia, $\geq$grade 3 infection or bleeding requiring transfusion, the dose in the next cycle was reduced by 25%. If the treatment day count showed an absolute granulocyte count of $<1.5\times10^9$/l or platelet count of $<100\times10^9$/l, treatment was delayed until recovery. Haematopoietic colony stimulating factors were not used. If patients developed grade 3 peripheral neuropathy, they were taken off protocol therapy, and a dose reduction of 25% was applied for grade 2 neurotoxicity. For other toxic effects $\geq$grade 3, drug administration was delayed until resolution to $\leq$grade 1, and then reinstituted with a dose reduction of 25%. Once doses had been reduced for toxicity, they were not re-escalated.

### *Patient evaluation*

Patients were evaluable for response after receiving one cycle of therapy (3 weeks on the study). Responding patients continued on the study until evidence of disease progression, or the occurrence of unacceptable toxicity. Patients with complete (CR) or partial (PR) response continued on therapy for a maximum of four cycles after documentation of response. Patients with stable disease (SD) continued on therapy for a maximum of six cycles. WHO criteria for response were employed and toxic effects were graded according to the NCIC-CTG common toxicity criteria.

### Results

Between April 1994 and June 1995, 32 patients were enrolled from eight Canadian centres. Two patients were ineligible: one had no measurable lesions and in the other the histology was not STS. Two further patients were not evaluable for response and haematological toxicity. The condition of one patient deteriorated suddenly with onset of congestive heart failure, renal failure and sepsis, and she died 6 days after the first dose of docetaxel. The second patient did not complete the first dose of docetaxel because of a severe hypersensitivity reaction and went off study. Table 1 describes the characteristics of eligible patients. A majority of patients (18) had lung metastases, other common sites of disease being nodes, soft tissue and within the abdomen/pelvis/retroperitoneum.

One hundred cycles were administered at the starting dose of 100 mg $m^{-2}$ every 3 weeks, and 26 cycles at the reduced dose level of 75 mg $m^{-2}$ every 3 weeks (a 25% dose reduction). One and five cycles were administered at dose levels 55 and $<50$ mg $m^{-2}$ respectively. The number of cycles per patient ranged from one to nine. The planned dose intensity was 33.3 mg $m^{-2}$ $week^{-1}$ and the achieved median dose intensity was 32.24 mg $m^{-2}$ $week^{-1}$ (range

**Table 1.** *Patient characteristics* (n = *30 eligible)*

| Characteristics | Number | |
|---|---|---|
| Sex | | |
| Female | 18 | |
| Male | 12 | |
| Performance status (ECOG) | | |
| 0 | 14 | |
| 1 | 11 | |
| 2 | 5 | |
| Prior therapy | | |
| Chemotherapy | 0 | |
| Radiotherapy | 14 | |
| Hormone therapy | 1 | |
| Immunotherapy | 0 | |
| Pathology | | |
| Leiomyosarcoma | 11 | |
| gastointestinal | | 6 |
| trunk | | 2 |
| uterine | | 3 |
| Malignant fibrous histiocytoma | 7 | |
| Mixed mullerian sarcoma | 2 | |
| Endometrial stromal sarcoma | 2 | |
| Haemangiopericytoma | 2 | |
| Undifferentiated | 2 | |
| Other* | 4 | |
| Number of sites of disease | | |
| 1 | 11 | |
| 2 | 14 | |
| 3 | 3 | |
| 4 or more | 2 | |

*Alveolar soft parts sarcoma, angiosarcoma, liposarcoma, malignant Schwannoma.

**Table 2.** *Haematological toxicity* (n = 28 evaluable)*

| Criteria | Median nadir (range) | Toxicity grade 0 | 1 | 2 | 3 | 4 |
|---|---|---|---|---|---|---|
| Haemoglobin (g/l) | 104 (62–149) | 5 | 11 | 8 | 3 | 1 |
| White cell count ($\times 10^9$/l) | 1.7 (0.7–13.9) | 3 | 2 | 5 | 12 | 6 |
| Granulocytes ($\times 10^9$/l) | 0.4 (0.2–7.5) | 6 | 1 | 1 | 2 | 18 |
| Platelets ($\times 10^9$/l) | 259 (136–564) | 26 | 2 | 0 | 0 | 0 |

*Worse by patient while on study.

**Table 3.** *Non-haematological toxicity—worst by patient* (n = 30 evaluable)*

| Type | Number by grade 1 | 2 | 3 | 4 | Incidence (%) |
|---|---|---|---|---|---|
| Alopecia | 13 | 8 | 2 | 0 | 77 |
| Lethargy | 8 | 6 | 4 | 0 | 60 |
| Nausea | 6 | 4 | 0 | 0 | 33 |
| Vomiting | 6 | 2 | 0 | 0 | 27 |
| Anorexia | 4 | 2 | 1 | 0 | 23 |
| Diarrhoea | 6 | 5 | 1 | 0 | 40 |
| Infection | 4 | 1 | 0 | 0 | 17 |
| Febrile neutropenia | 0 | 0 | 7 | 0 | 23 |
| Fever (no infection) | 3 | 1 | 0 | 0 | 13 |
| Dyspnoea | 1 | 1 | 0 | 1 | 10 |
| Oedema | 4 | 2 | 2 | 0 | 27 |
| Myalgia | 1 | 0 | 2 | 0 | 10 |
| Arthralgia | 2 | 0 | 1 | 0 | 10 |
| Peripheral neuropathy—motor | 3 | 1 | 1 | 0 | 17 |
| Peripheral neuropathy—sensory | 9 | 4 | 0 | 0 | 43 |

*Felt to be drug related.

1.25–33.60 mg $m^{-2}$ $week^{-1}$). Sixty-seven per-cent of patients were able to receive $\geq$90% of the planned dose intensity.

Myelosuppression could be evaluated in 28 patients having interim blood counts. Neutropenia was common and moderately severe, 18 (64%) patients experiencing at least one episode of grade 4 neutropenia (Table 2). Febrile neutropenia was reported in seven (25%) patients. Two other patients had a documented grade 4 infection not thought to be related to therapy. In 30 evaluable patients, the most frequently observed non-haematological toxicities thought to be drug related were alopecia (77%), lethargy (60%), sensory neuropathy (43%), oedema (27%), nausea (33%), vomiting (27%), anorexia (23%) and diarrhoea (40%), but most of these were mild (Table 3).

In 28 evaluable patients, there were three PRs (10.7%; 95% confidence interval (CI) 2.3–28.2%) with a median duration of 7 months (range 6.4–8.3 months). Thirteen patients had lasting 5.4 months (range 2.1–20.9 months). The median number of cycles received by these patients was six (range 3–14). Two of the PRs occurred in patients with leiomyosarcoma (one uterine, one retroperitoneal), the most common histological type entered into the study. The third response occurred in a patient with malignant fibrous histiocytoma. Two of the responses occurred in lung metastases, and the third in a retroperitoneal mass.

### Discussion

The taxanes, paclitaxel and docetaxel, are a new class of cytotoxic drugs with activity in a variety of solid tumours. Paclitaxel was the first analogue to be tested, and was shown to be active in platinum-resistant ovarian cancer[9] and also in breast cancer.[10] A difficult extraction process, from the bark of the Pacific yew, initially limited supplies and accelerated the search for semi-synthetic analogues, such as docetaxel.

In phase II trials, docetaxel has shown significant activity, with reproducible response rates of greater than 20%, in breast cancer, non-small cell lung, ovarian, gastric, squamous head and neck and bladder cancers.[11]

Both analogues have been evaluated in advanced/metastatic STS. There have been two studies of paclitaxel at a dose of 250 mg $m^{-2}$ every 3 weeks. In the larger South West Oncology Group (SWOG) study[12], there was one CR and 5 PRs for an overall response rate of 12.5% (95% CI 4.7–25.3%), in 48 patients none of whom had received prior chemotherapy. A lower response rate of one PR (3.6%) in 28 patients, 11 of whom had received prior chemotherapy, was reported by Waltzman *et al.*[13] Gian *et al.*[14] did not observe any responses in 10 patients with STS (four had received prior chemotherapy) treated with 200 mg $m^{-2}$ every 3 weeks of paclitaxel.

In contrast, Van Hoesel *et al.*,[15] for the European Organization for Research and Treatment of Cancer (EORTC), observed five PRs in 29 patients (17.2%; 95% CI 6–36%) treated with docetaxel 100 mg $m^{-2}$ every 3 weeks, all of whom had received previous chemotherapy. These encouraging results prompted the EORTC to undertake a randomized phase III study comparing 100 mg $m^{-2}$ every 3 weeks of docetaxel with 75 mg $m^{-2}$ every 3 weeks of doxorubicin. This study has just been reported in abstract form.[16] With 86 patients on study and 73 available for analysis, the preliminary response rates were 0% for docetaxel and 27% for doxorubicin, the latter being very similar to previous EORTC experience with single-agent doxorubicin.

The current study was started shortly after publication in abstract form[17] of the preliminary results of the EORTC phase II study describing, at that time, five PRs (21.7%) in 23 patients. We hoped to confirm, and perhaps improve on, these results in chemotherapy-naïve patients. The low response rate seen in our study was surprising, but is less so now that it is seen in the context of the more recent EORTC phase III results.[16] It is intriguing that in a different tumour type, small-cell lung cancer, a higher response rate of 25%[18] was seen in previously treated patients receiving docetaxel, compared with 8%[19] in a second study including only chemotherapy-naïve patients. As the EORTC study[17] includes a cross-over component this may provide further data on this issue.

Thirteen patients achieved SD. Rapidly progressive disease at entry was not a protocol requirement, but most Canadian oncologists use chemotherapy in the setting of progressive symptomatic metastatic disease, and this disease stabilization may be an additional indicator of drug activity.

It has been suggested that leiomyosarcomas, particularly those of bowel metastatic to liver, are less responsive to chemotherapy. However, leiomyosarcomas (including those of gastrointestinal origin now known as stromal tumours) may comprise one-third to one-half of tumours in clinical trials of metastatic STS. This is true of our study (39%), the phase II[15] and III[17] studies of the EORTC (41%; 36%) and for the SWOG[12] and Waltzman studies[13] of paclitaxel, 54% and 53% respectively. This similar distribution does not provide a reason for the discrepant response rates. The most likely explanation is chance occurrence in small patient populations.

### Conclusions

Despite initial promise, data from this study, and more recent phase II and III studies, do not suggest that taxanes have major activity in adult STS.

### Acknowledgements

A study of the National Cancer Institute of Canada Clinical Trials Group and the Canadian Sarcoma Group. We would like to thank W. Bogues, D. Charpentier, J. Latrielle, F. Letendre, J. Jensen, M. Knowling, D. Stein, M. Thirlwell and L. Yelle who also contributed patients to this study. Supported by a grant from Rhône-Poulenc-Rorer Ltd.

### References

1 Van Geel AN, Pastorino U, Jauch KW, *et al.* Surgical treatment of lung metastases. The European Organization for Research and Treatment of Cancer—Soft Tissue and Bone Sarcoma Group study of 255 patients. *Cancer* 1996; 77:675–82.

2 Bramwell VHC. Chemotherapy for metastatic soft tissue sarcomas—another full circle? *Br J Cancer* 1991; 64:7–9.

3 Santoro A, Tursz T, Mouridsen H, *et al.* A. Doxorubicin versus CYVADIC versus doxorubicin plus ifosfamide in first-line treatment of advanced soft tissue sarcomas:a randomized study of the European Organization for Research and Treatment of Cancer Soft Tissue and Bone Sarcoma Group. *J Clin Oncol* 1995; 13:1537–45.

4 Tursz T, Verweij J, Judson I, *et al.* Is high-dose chemotherapy of interest in advanced soft tissue sarcomas (ASTS)? An EORTC randomized phase III trial. *Proc Am Soc Clin Oncol* 1996; 15:337.

5 Lavelle F, Fizames C, Gueritte-Voegelin F, *et al.* Experimental properties of RP56976, a taxol derivative. *Proc Am Assoc Cancer Res* 1989; 30:566.

6 Ringel I, Horwitz SB. Studies with RP56976 (docetaxel): a semisynthetic analogue of taxol. *J Natl Cancer Inst* 1991; 83:288–91.

7 Bissery MC, Bayssas M, Lavelle F. Preclinical evaluation of intravenous docetaxel RP56976, NSC 628503), a taxol analog. *Proc Am Assoc Cancer Res* 1990; 31:417.

8 Extra J-M, Rousseau F, Bruno R, *et al.* Phase I and pharmacokinetic study of Taxotere (RP56976; NSC 628503) given as a short intravenous infusion. *Cancer Res* 1993; 53:37–42.

9 McGuire WP, Rowinski EK, Rosenhein NB, *et al.* Taxol, a unique antineoplastic agent with significant activity in advanced ovarian epithelial neoplasms. *Ann Intern Med* 1989; 111:273–9.

10 Holmes FA, Frye D, Theriault HL, *et al.* Phase II study of taxol in patients with metastatic breast cancer. *Proc Am Soc Clin Oncol* 1991; 113.

11 Eisenhauer EA. Docetaxel: current status and future prospects. *J Clin Oncol* 1995; 13:2865–8.

12 Balcerzak SP, Benedetti J, Weiss GR, *et al.* A phase II trial of paclitaxel in patients with advanced soft tissue sarcomas. A Southwest Oncology Group study. *Cancer* 1995; 76:2248–52.

13 Waltzman R, Schwartz GK, Shorter S, *et al*. Lack of efficacy of paclitaxel (Taxol) in patients with advanced soft tissue sarcoma (soft tissue sarcoma). *Proc Am Soc Clin Oncol* 1996; 15:526.

14 Gian V, Lynch J, McCarley D, *et al*. Taxol in the treatment of recurrent or metastatic soft tissue or osteosarcomas. *Proc Am Soc Clin Oncol* 1995; 14:516.

15 Van Hoesel QGCM, Verweij J, Catimel G, *et al*. Phase II study with docetaxel (Taxotere) in advanced soft tissue sarcomas of the adult. *Ann Oncol* 1994; 5:539–42.

16 Verweij J, Judson I, Crowther D, *et al*. Randomized study comparing docetaxel (Taxotere) to doxorubicin (D) in previously untreated soft tissue sarcomas (STS). *Proc Am Soc Clin Oncol* 1997; 16:496.

17 Van Hoesel OGCM, Verweij J, Clavel M, *et al*. Phase II study with Taxotere (RP56976) in advanced soft tissue sarcomas of the adult. *Eur J Cancer* 1993; 29A:183.

18 Smyth JF, Smith IE, Sessa C, *et al*. Activity of docetaxel (Taxotere) in small cell lung cancer. *Eur J Cancer* 1994; 30A:1058–60.

19 Latreille J, Cormier Y, Martins H, *et al*. Phase II study of docetaxel (Taxotere) in patients with previously untreated extensive small cell lung cancer. *Invest New Drugs* 1996; 13:343–5.

## References

[PDF_00444] Balcerzak S. P., Benedetti J., Weiss G. R., Natale R. B. (1995). A phase II trial of paclitaxel in patients with advanced soft tissue sarcomas. A Southwest Oncology Group study.. Cancer.

[PDF_00407] Bramwell V. H. (1991). Chemotherapy for metastatic soft tissue sarcomas--another full circle?. Br J Cancer.

[PDF_00442] Eisenhauer E. (1995). Docetaxel: current status and future prospects.. J Clin Oncol.

[PDF_00471] Latreille J., Cormier Y., Martins H., Goss G., Fisher B., Eisenhauer E. A. (1996). Phase II study of docetaxel (taxotere) in patients with previously untreated extensive small cell lung cancer.. Invest New Drugs.

[PDF_00435] McGuire W. P., Rowinsky E. K., Rosenshein N. B., Grumbine F. C., Ettinger D. S., Armstrong D. K., Donehower R. C. (1989). Taxol: a unique antineoplastic agent with significant activity in advanced ovarian epithelial neoplasms.. Ann Intern Med.

[PDF_00424] Ringel I., Horwitz S. B. (1991). Studies with RP 56976 (taxotere): a semisynthetic analogue of taxol.. J Natl Cancer Inst.

[PDF_00410] Santoro A., Tursz T., Mouridsen H., Verweij J., Steward W., Somers R., Buesa J., Casali P., Spooner D., Rankin E. (1995). Doxorubicin versus CYVADIC versus doxorubicin plus ifosfamide in first-line treatment of advanced soft tissue sarcomas: a randomized study of the European Organization for Research and Treatment of Cancer Soft Tissue and Bone Sarcoma Group.. J Clin Oncol.

[PDF_00468] Smyth J. F., Smith I. E., Sessa C., Schoffski P., Wanders J., Franklin H., Kaye S. B. (1994). Activity of docetaxel (Taxotere) in small cell lung cancer. The Early Clinical Trials Group of the EORTC.. Eur J Cancer.

[PDF_00402] van Geel A. N., Pastorino U., Jauch K. W., Judson I. R., van Coevorden F., Buesa J. M., Nielsen O. S., Boudinet A., Tursz T., Schmitz P. I. (1996). Surgical treatment of lung metastases: The European Organization for Research and Treatment of Cancer-Soft Tissue and Bone Sarcoma Group study of 255 patients.. Cancer.

[PDF_00456] van Hoesel Q. G., Verweij J., Catimel G., Clavel M., Kerbrat P., van Oosterom A. T., Kerger J., Tursz T., van Glabbeke M., van Pottelsberghe C. (1994). Phase II study with docetaxel (Taxotere) in advanced soft tissue sarcomas of the adult. EORTC Soft Tissue and Bone Sarcoma Group.. Ann Oncol.

